# Mechanisms Underlying the Immune Response Generated by an Oral *Vibrio cholerae* Vaccine

**DOI:** 10.3390/ijms17071062

**Published:** 2016-07-02

**Authors:** Danylo Sirskyj, Ashok Kumar, Ali Azizi

**Affiliations:** 1 Department of Biochemistry, Microbiology and Immunology, Faculty of Medicine, University of Ottawa, Ottawa, ON K1H 8M5, Canada; dsirskyj@gmail.com (D.S.); akumar@uottawa.ca (A.K.); 2 Children’s Hospital of Eastern Ontario (CHEO)-Research Institute, Ottawa, ON K1H 5B2, Canada; 3 Department of Pathology and Laboratory Medicine, University of Ottawa, 451 Smyth Rd, Ottawa, ON K1H 8M5, Canada

**Keywords:** oral vaccine, Toll-like receptor, humoral immunity, *Vibrio cholerae*

## Abstract

Mechanistic details underlying the resulting protective immune response generated by mucosal vaccines remain largely unknown. We investigated the involvement of Toll-like receptor signaling in the induction of humoral immune responses following oral immunization with Dukoral, comparing wild type mice with TLR-2-, TLR-4-, MyD88- and Trif-deficient mice. Although all groups generated similar levels of IgG antibodies, the proliferation of CD4+ T-cells in response to *V. cholerae* was shown to be mediated via MyD88/TLR signaling, and independently of Trif signaling. The results demonstrate differential requirements for generation of immune responses. These results also suggest that TLR pathways may be modulators of the quality of immune response elicited by the Dukoral vaccine. Determining the critical signaling pathways involved in the induction of immune response to this vaccine would be beneficial, and could contribute to more precisely-designed versions of other oral vaccines in the future.

## 1. Toll-Like Receptors and Dukoral Vaccine

The mechanisms responsible for the induction of protective immune responses from mucosal vaccines are not yet clear. Since most infections start at mucosal surfaces, an understanding of the precise mechanistic and signaling details underlying a licensed mucosal vaccine could assist in the development of new vaccines [1,2,3]. Dukoral is an orally administered vaccine, to protect against travellers’ diarrhea caused by ETEC, as well as for the prevention of cholera, caused by *Vibrio cholera* [4,5]. The Dukoral vaccine is containing *V. cholerae* (comprised of heat-inactivated *V. cholerae* 01 Inaba classic strain and Ogawa classic strain, and formalin-inactivated *V. cholerae* 01El Tor strain and Ogawa classic strain) along with the recombinant cholera toxin B-subunit protein (CTB) [4,5]. Included with the vaccine is a bicarbonate buffer to be ingested together with the vaccine at the time of immunization, for the purpose of neutralizing residual stomach acid in order to protect the integrity of the vaccine antigens. Mechanistic details on precise immune pathways involved in the induction of an immune response to this vaccine are largely lacking from the product monograph.

Early immune responses against invading pathogens occur through the sensing of multiple microbial structures [6] by receptors, including Toll-like receptors (TLRs) [7]. TLRs are expressed predominantly on monocytes/macrophages, dendritic cells (DCs), B-cells, and T-cells [8,9,10,11,12,13]. All TLRs, excluding TLR-3, utilize the MyD88 signaling adaptor to induce the production of proinflammatory cytokines by way of the NF-κB and other transcription factors [14,15,16]. Alternatively, TLR-4 has been shown to utilize the MyD88 and Trif signaling adaptors, in order to induce the production of proinflammatory cytokines and type I interferons by way of NF-κB and interferon regulatory factor 3 (IRF-3) [16]. Trif has been described as such a signaling adaptor molecule in the MyD88-independent signaling pathway, stimulated by TLR-3 and TLR-4 [17,18].

To date, the precise requirement of TLR signaling for the generation of protective antibody responses to antigens from licensed commercial vaccines is not clear. Our group began uncovering such mechanistic details, by examining the requirement of TLR signaling (specifically, the requirement of MyD88, Trif, TLR-2 and TLR-4 signaling) in the induction of immune responses in mice, following immunization with the Dukoral vaccine. All subsequent animal studies utilized female mice of the C57BL/6 background, between 7 and 10 weeks of age (Jackson Laboratory, Bar Harbor, ME, USA). At the time these experiments were carried out additional mutants were only available on the Balb/C background. As such, to ensure uniformity of the experimental results and to ensure that all animals were of the same genetic background, groups of TLR-2-, TLR-4-, MyD88-, Trif-deficient mice, as well as wild type mice, were investigated. Mutant strains were described by the supplier as having large deletions of their respective TLR gene which eliminated the expression of TLR mRNA and corresponding protein. To evaluate the precise mechanistic underlying the immune responses generated by the Dukoral vaccine, the role of other TLRs (using TLR-deficient mice with the same genotype) should be also investigated.

In this study, animals were housed in micro-isolator cages under specific-pathogen free conditions, with food and water provided ad libitum. All mice being orally immunized were fasted for at least 4 h prior to immunization, and first received a 100 μL dose of sodium hydrogen carbonate buffer to neutralize residual stomach acid. All neutralizing buffer and vaccine doses were administered intra-gastrically by gavage needle. To determine the optimal oral dose of the Dukoral vaccine in animals, C57BL/6 WT mice were orally immunized with various amounts of *V. cholerae* along with 10 µg CTB, on days 0, 10, 20 and 30. Serum and feces were collected before and after each immunization. C57BL/6 mice receiving 3 × 10^9^ *V. cholerae* with 10 µg CTB showed the highest *V. cholerae* and CTB-specific serum IgG and fecal IgA responses. Since this dose of vaccine resulted in the highest *V. cholerae* specific serum and fecal antibody responses, four oral immunizations with 3 × 10^9^ *V. cholerae* and 10 µg CTB were found to be the optimal oral dose of Dukoral vaccine for C57BL/6 mice (data not shown). Oral vaccine doses were administered in a 100 μL volume. TLR mutant mice and WT controls (*n* = 5 mice per group) were orally immunized on days 0, 10, 20, and 30, with 3 × 10^9^ *V. cholerae* and 10 µg CTB.

Pre- and post-vaccination sera and feces were collected. Blood was collected via saphenous vein puncture, centrifuged to obtain serum, and stored at −20 °C until used. Fecal pellets were collected and stored at −80 °C prior to use. To extract fecal antibody, 100 mg of feces per mouse was weighted out, then dissolved in 1 mL PBS containing 2.5% non-fat milk with complete mini EDTA-free protease inhibitors (Roche Applied Science, Laval, QC, Canada). Fecal pellets were broken up using a pipette tip, vortexed, and incubated on ice for 1 h with intermittent vortexing. Next, samples were centrifuged for 15 min at 4 °C to pellet debris. The supernatant was then collected and stored at −80 °C until analyzed.

**Figure 1:**
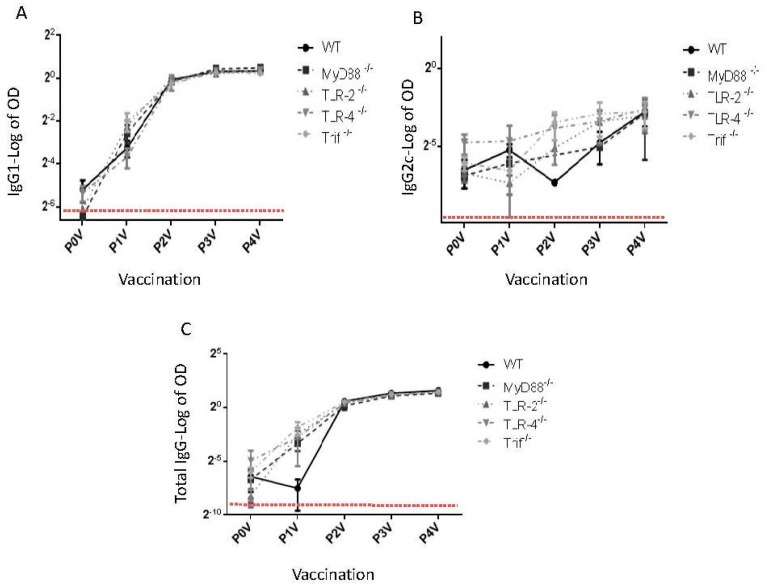
TLR signaling is dispensable for *V. cholerae*-specific antibody production following oral immunization with Dukoral vaccine. TLR mutant mice and WT controls (*n* = 5 mice per group) were orally immunized on days 0, 10, 20, and 30, with 3 × 10^9^ *V. cholerae* and 10 µg CTB. Serum was collected 9 days after each vaccination and *V. cholerae*-specific antibody titer against **A**, IgG1, **B**, IgG2a, and **C**, IgG-Fc was measured by ELISA. The plates were coated with 50 μL of 12 × 10^6^ *V. cholerae*/mL. The next day, coating buffer was decanted and plates were washed once in PBS. Plates were then blocked for 2 h at 37 °C with 2% FCS-PBS. After blocking, plates were washed once in PBS and diluted samples were added to duplicate wells. After incubating for 1 h at 37 °C, plates were washed with PBS and then incubated for 1 h at 37 °C with 50 μL of secondary horseradish peroxidase (HRP)-conjugated secondary antibody in 2% FCS-PBS (goat anti-mouse A. IgG1, B. IgG2a, C. IgG-Fc). Plates were then washed and developed with 3,3′,5,5′-tetramethylbenzidine (TMB) one component HRP microwell substrate. The reaction was stopped by stop solution and plates were read on at 450 nm. The naïve control data are shown as dashed line. Results are shown as the mean log of O.D 450 nm ± SEM. P0V denotes pre-vaccination serum. P1V−P4V denotes post-1st vaccination through post-4th vaccination time points. The production of IgG, IgG1, and IgG2c was not statistically significant (*p* ≥ 0.05).

None of the TLR-deficient animals (MyD88^−/−^, Trif^−/−^, TLR-2^−/−^, and TLR-4^−/−^) showed any significant impairment in the generation of *V. cholerae*-specific or CTB-specific serum antibodies compared to those generated in WT mice, at any time following immunization. The *V. cholerae*-specific antibody titers following various immunizations are shown in Figure 1. These findings suggest that TLR signaling may not be essential for the induction of IgG in sera.

**Figure 2:**
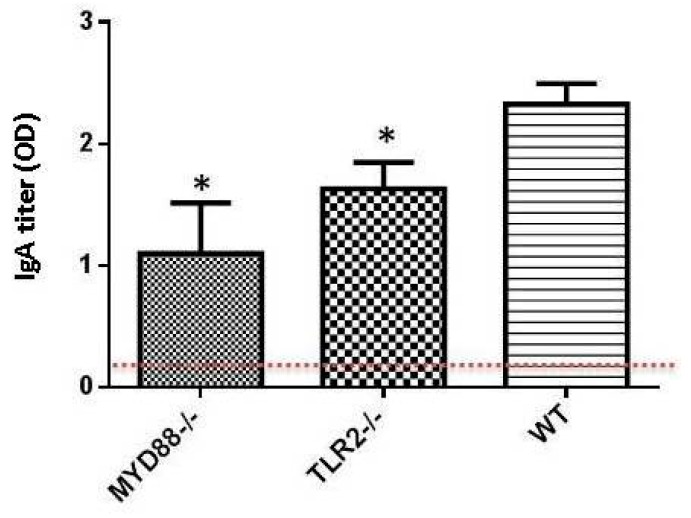
TLR signaling might mediate the generation of CTB-specific fecal IgA antibody production. TLR mutant (TLR-2^−/−^, TLR-4^−/−^, MyD88^−/−^ and Trif^−/−^) and WT controls (*n* = 5 per group) were immunized orally on days 0, 10, 20, and 30 with Dukoral (3 × 10^9^ *V. cholerae* with 10 μg CTB). Fecal pellets were collected pre-vaccination and 9 days after the last vaccination. Fecal supernatants were extracted and fecal CTB-specific IgA antibodies were measured by ELISA at the indicated dilutions. Results shown are the post-4th vaccination mean O.D 450 nm ± SEM. * *p* ≤ 0.05. The naïve control data are shown as dashed line.

IgA is the predominant antibody on mucosal surfaces and is the primary line of protection against pathogens. Therefore, the requirement of TLR signaling for the generation of fecal antigen-specific IgA antibody was investigated. Fecal samples from orally immunized mice were collected and tested for the presence of vaccine antigen-specific IgA antibody responses. For measuring fecal IgA antibody responses, fecal pellets was processed and 50 μL of clarified fecal supernatant at an undiluted, 1:10, and 1:100 dilution was applied to antigen-coated plates. While none of the groups of TLR-deficient animals showed significantly impaired *V. cholerae*-specific fecal IgA antibody production (data not shown), MyD88^−/−^ and TLR-2^−/−^ mice were significantly impaired in their ability to produce CTB-specific fecal IgA (*p* = 0.02 and *p* = 0.03 respectively) (Figure 2). These results suggest that TLR signaling may be required for the generation of CTB-specific IgA but not *V. cholerae*-specific IgA antibodies.

To study the involvement of TLR signaling in the generation of protection by the vaccine, serum antibodies were evaluated using an in vitro agglutination assay using live *V. cholerae*. Samples were tested against three 01 Inaba strains, two 01 Ogawa strains, and one 01 Inaba El Tor strain, for a total of six strains. The agglutination assay was performed in the National Microbiology Laboratory at Public Health Agency of Canada (Winnipeg, MB, Canada). The results demonstrated that serum from TLR-2^−/−^ and TLR-4^−/−^ animals was unable to agglutinate live *V. cholerae*, despite those animals being unimpaired in their ability to generate *V. cholerae* or CTB specific serum IgG antibodies. Surprisingly, serum from immunized MyD88^−/−^ and Trif^−/−^ mice was able to induce *V. cholerae* agglutination. While it is not yet clear how MyD88^−/−^ mice showed agglutination to live *V. cholerae* while TLR-2^−/−^ and TLR-4^−/−^ animals did not, we assume that the Dukoral vaccine might activate additional receptors or other TLRs (e.g., TLR1, TLR5, TLR6, TLR10), subsequently activating non-MyD88 pathways (data not shown). Due to the limitations in the volume of serum collected, the agglutination assay could only be performed once. Additional experiments should be executed to confirm the obtained data.

**Figure 3:**
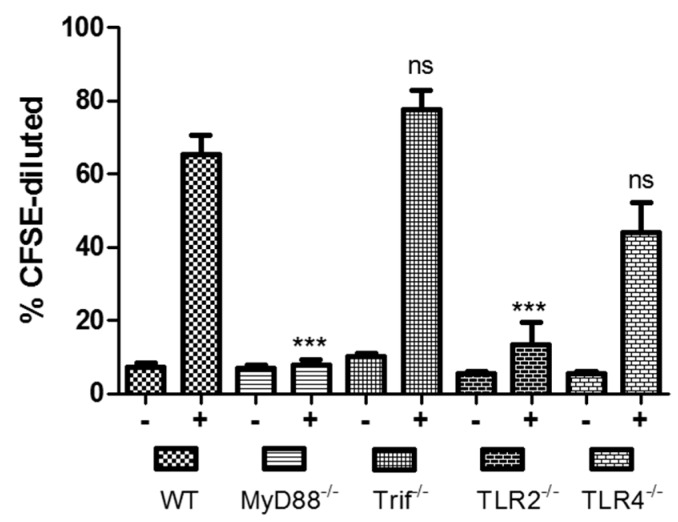
CD4+ T-cell proliferation in response to stimulation by *V. cholerae*. Splenocytes from immunized mice were collected 2 weeks after the last vaccination, labelled with 5 µM CFSE and stimulated with *V. cholerae* for 5 days. Proliferation was evaluated by flow cytometry by the degree of CFSE-dilution on fluorescently-labelled CD4+ T-cells. Cell proliferation is shown as the % of CFSE-diluted events from 10,000 gated events ± SEM. −, unstimulated; +, stimulated with *V. cholerae* at a ratio of 1:2000. *** *p* < 0.001, ns, not significant.

To evaluate CD4+ T-cell immune responses, spleens from immunized animals were collected 2 weeks after the last vaccination, labelled with 5 µM CFSE, stimulated with *V. cholerae* for 5 days, and proliferation was evaluated by flow cytometry by the degree of CFSE-dilution on fluorescently-labelled CD4+ T-cells. The results show that CD4+ T-cells from orally immunized TLR-2^−/−^ (*p* = 0.0002) and MyD88^−/−^ (*p* < 0.0001) mice were significantly inhibited in their ability to proliferate in response to stimulation with whole-cell *V. cholera*, compared to wild type control mice (Figure 3). Proliferation of CD4+ T-cells from TLR-4^−/−^ mice was also decreased, but not significantly. No inhibition of proliferation was seen in CD4+ T-cells from Trif^−/−^ mice, suggesting that CD4+ T cell proliferation in response to *V. cholerae* stimulation occurs independently of Trif signaling.

## 2. Conclusions

In summary, our results indicate that TLR pathways may have important roles in regulating the quality of immune response induced following oral immunization with the Dukoral vaccine. The results also suggest that proliferation of both CD4+ T-cells in response to *V. cholerae* is mediated via MyD88/TLR signaling, and independently of Trif signaling. Our results represent novel findings regarding the role of TLR signaling in the induction of humoral immune responses to the Dukoral vaccine.

## Abbreviations

CTB	Cholera toxin B subunit
TLR	Toll-Like Receptors
ETEC	Enterotoxigenic *Escherichia coli*
*V. cholera*	*Vibrio cholera*
